# A guide to the Proteomics Identifications Database proteomics data repository

**DOI:** 10.1002/pmic.200900402

**Published:** 2009-09

**Authors:** Juan Antonio Vizcaíno, Richard Côté, Florian Reisinger, Joseph M. Foster, Michael Mueller, Jonathan Rameseder, Henning Hermjakob, Lennart Martens

**Affiliations:** 1 EMBL Outstation, European Bioinformatics Institute, Wellcome Trust Genome CampusHinxton, Cambridge, UK; 2 Computational and Systems Biology Initiative, Massachusetts Institute of TechnologyCambridge, MA, USA

**Keywords:** Bioinformatics, Data repository, Mass spectrometry

## Abstract

The Proteomics Identifications Database (PRIDE, http://www.ebi.ac.uk/pride) is one of the main repositories of MS derived proteomics data. Here, we point out the main functionalities of PRIDE both as a submission repository and as a source for proteomics data. We describe the main features for data retrieval and visualization available through the PRIDE web and BioMart interfaces. We also highlight the mechanism by which tailored queries in the BioMart can join PRIDE to other resources such as Reactome, Ensembl or UniProt to execute extremely powerful across-domain queries. We then present the latest improvements in the PRIDE submission process, using the new easy-to-use, platform-independent graphical user interface submission tool PRIDE Converter. Finally, we speak about future plans and the role of PRIDE in the ProteomExchange consortium.

## 1 Introduction

Bioinformatics tools and data repositories provide one of the main pillars of biology in the 21st century. Indeed, public availability of biological data *via* the Internet has changed the way biologists plan, execute and interpret their studies. Some of the best known protein-related resources include UniProt 1 for protein sequences and annotation, the Protein Databank 2 and other members of the wwPDB consortium 3 for protein structures, Intact 4 and other components of the IMEx consortium 5 for protein interactions, InterPro 6 for protein domains, and UniMod 7 and RESID 8 for protein modifications.

Like in any other “omics” field, the amount of data generated by MS based proteomics has increased exponentially in the last few years, which prompted the development of several data repositories. At the same time, proteome informatics efforts have driven the development of universally adopted and stable data formats under the auspices of the HUPO Proteomics Standards Initiative (HUPO-PSI, http://www.psidev.info), and have led to powerful data analysis strategies 9,10. Taken together, these advances have allowed the centralized aggregation of proteomics data and its reanalysis or meta-analysis, ultimately turning proteomics into a much more robust discipline in the life sciences.

Several proteomics MS data repositories have been established so far, with GPMDB 11, Proteomics Identifications Database (PRIDE) 12, PeptideAtlas 13 and Proteinpedia 14 among the most prominent ones at present 15,16. Additionally, the NCBI recently launched their Peptidome (http://www.ncbi.nlm.nih.gov/projects/peptidome) system as a centralized, public proteomics repository not dissimilar from PRIDE. The Tranche (http://tranche.proteomecommons.org) system is used in the field as well, and essentially presents a data transfer layer relying on peer-to-peer Internet protocol technology. Apart from these large-scale efforts, there are also smaller, more specialized repositories such as PepSeeker 17, the Genome Annotating Proteomic Pipeline 18 or MAPU 19. Many of these repositories have different aims and therefore offer different functionalities. For an up to date review covering the capabilities of these different proteomic MS repositories see 16.

Together with the newly released NCBI Peptidome, the established PRIDE (PRoteomics IDEntifications) database at the European Bioinformatics Institute (EBI, http://www.ebi.ac.uk/pride) occupies a special place in the list of proteomics resources, in that it constitutes an actual structured data repository, and does not assume editorial control over submitted data. Moreover, several highly influential informatics tools have been developed in support of the PRIDE database: the Ontology Lookup Service (OLS, http://www.ebi.ac.uk/ols) 20,21, the Protein Identifier Cross-Referencing system (PICR, http://www.ebi.ac.uk/Tools/picr) 22, and Database on Demand (http://www.ebi.ac.uk/pride/dod) 23. Several data submission tools are available for PRIDE, including the powerful and popular PRIDE Converter (http://code.google.com/p/pride-converter) 24.

Here we will explain how the current PRIDE system works and how potential users can use PRIDE and its satellite tools for both data retrieval and data submission. Finally, we will discuss some of the future improvements that are planned, including the exchange of data with other repositories.

## 2 The PRIDE system and its associated tools OLS and PICR

The PRIDE project started at the EBI in 2003 as a Marie Curie fellowship, and the first production system (v 1.0) was online and populated with data by the end of 2004 25. By that time, the need for data sharing through centralized repositories in proteomics had already been pointed out 26. Since that moment, PRIDE has grown in step with the field and has become one of the most important public proteomics resources 12,27. Because data in PRIDE is not reprocessed or altered in any way after submission, and because PRIDE allows data to remain private while anonymously sharing it with journal editors and reviewers, PRIDE is now the recommended submission point for several journals such as *Nature Biotechnology* 28, *Nature Methods* 29, and *Proteomics* (http://www3.interscience.wiley.com/cgi-bin/jabout/76510741/2120_instruc.pdf).

Since its inception, PRIDE has always committed to use community data standards as formulated by the HUPO-PSI. Currently implementing the mzData standard (http://www.psidev.info/index.php?q=node/80), PRIDE will adopt the emerging mzML (for MS, http://www.psidev.info/index.php?q=node/80) and mzIdentML (known before as analysisXML, for protein and peptide identification, http://www.psidev.info/index.php?q=node/319) data formats when they are released. Both formats are currently being refined through the PSI document process and are expected to be finalized by autumn 2009.

The PRIDE system is developed in Java and is available in full as open source under the permissive Apache2 license (http://code.google.com/p/ebi-pride/). As a result, it is easy to set up a local installation of PRIDE if desired. Further documentation and guidance for software engineers and bioinformaticians wishing to deploy local installations of PRIDE are provided on the above mentioned website. Furthermore, technical support is always available through pride-support@ebi.ac.uk.

PRIDE stores three different kinds of information: peptide and protein identifications derived from MS or MS/MS experiments, MS and MS/MS mass spectra as peak lists, and any and all associated metadata. Experiments constitute the basic unit of information and at the moment of writing, PRIDE contains around 9700 experiments, containing more than 2.2 million protein identifications supported by 10.7 million peptides, based on more than 47 million mass spectra.

PRIDE also has the concept of a project, which is a way to organize different related experiments together in a hierarchical structure. It is not mandatory to provide a project name when you are submitting related experiments to PRIDE but it is highly recommended, as it will allow efficient retrieval of data across the different related experiments.

As mentioned before, PRIDE relies heavily on two additional tools: OLS and PICR. These services are also very popular in their own right, and both direct web interfaces as well as programmatic web services are provided to interact with these tools. OLS provides convenient and powerful access to a large number of biomedical ontologies and controlled vocabularies 21. PRIDE takes advantage of OLS to store, structure, and present any and all metadata annotations on experiments, proteins, peptides and mass spectra. The extensive use of controlled vocabularies and ontologies for flexible yet context-sensitive annotation of data, along with the ability to perform intelligent queries by these annotations, are unique features that currently set PRIDE apart from any other proteomics repository.

The PICR tool on the other hand, is built to overcome one of the most infamous problems in proteomics: the existence of heterogeneous and changing identifiers or accession numbers referring to the same protein in different databases 22. PICR is used to map all the submitted protein identifications in PRIDE to all known accession numbers (including older accession numbers that are no longer in use) for those proteins in the most important protein databases (including UniProt 30, IPI 31, Ensembl 32, and RefSeq 33, but also to some genomic databases such as WormBase 34 and FlyBase 35). Protein identifications in PRIDE that were originally derived from different databases, or from different time points of the same database, thus become fully comparable. These PICR mappings are performed on the entire PRIDE database at regular intervals in order to keep all mappings up-to-date.

## 3 PRIDE web interface

The PRIDE web interface includes all necessary pages and forms to provide the user with access to its core functionality. Detailed, step-by-step usage of these pages has been described extensively before 36,37. Here, we present an overview of the main features of the web interface.

Apart from the “PRIDE Basic Statistics” and “Page Contents” boxes, both located at the top right of the main page, the main functionality resides in the menu on the left of the page. The “Search PRIDE” box, on the top left, can be used to query PRIDE with experiment accession numbers, protein accession numbers from different databases (which automatically includes a search of the PICR mappings), and terms from the taxonomy-related NEWT ontology (for instance NEWT:9606, for data from human samples).

However, if more complex queries are needed, several other options are possible:

1. The “Advanced Search” form allows the user to query PRIDE by protein identification accession number, peptide sequence, bibliographic reference, or sample details (including species, tissue, and cell type).
2. The “Browse Experiments” page includes direct access to PRIDE experiments organized by project (top of page), or by sample properties such as species, cell type, GO ontology, and phenotype (bottom of page). The latter search by sample annotation is quite powerful as it relies on OLS to search not only on an exact term match but also on a match to any child term of the selected term. For instance, a search on tissue term “brain” will also return datasets annotated as “cerebral cortex,” as the latter is specified in the corresponding BRENDA Tissue Ontology to be “a part of” brain, and is therefore automatically included in the actual query executed by PRIDE.
3. The “PRIDE BioMart” interface (see below).

Once a simple or complex query has been performed, you will be taken to the “Search Summary View.” This form provides an overview of the datasets that matched the query, and provides several options for further investigating the individual experiments in detail. This view can also be used to compare protein identifications found in up to ten experiments, with the results displayed as a Venn diagram (if you are comparing two or three experiments) or a histogram (for comparisons of more than three experiments). Clicking any section of the resulting diagram will display a list of the proteins present in that section. It is important to note that the experiment comparison makes full use of the PICR mappings in PRIDE to perform the most robust and comprehensive comparison possible. This ability to compare heterogeneous datasets (obtained from different databases, and/or on different times) is another unique feature of PRIDE.

From the “Search Summary View,” each experiment can either be displayed in detail in an HTML page, or can be downloaded as a zipped PRIDE XML formatted data file. If the second choice is chosen, three options are available: an XML file containing “Identifications and Spectra” (all data in the experiment), “Identifications only,” or “Spectra only” (in mzData format). All these files are also directly available from the EBI FTP server (ftp://ftp.ebi.ac.uk/pub/databases/pride). The only exception is for experiments that have been submitted without mass spectra where no “Spectra only” mzData version is made available.

In the “Experiment View” page, all the available information about the experiment is displayed including contact details, sample, and protocol details, and importantly, prominent links to the original paper references. Further down on this page are links to the “identification” and “spectrum” information. Additionally, there is a direct link to visualize the identified proteins in the experiment on the Reactome “Sky Painter.” The Reactome database is a curated resource for human pathway data 38, and the Sky Painter provides a graphical interface that shows all currently annotated pathways in Reactome. The Sky Painter link thus allows PRIDE users to find out which pathways are represented by the proteins from a particular experiment.

If the user next chooses to go to the “spectrum” page, all the information about the instrument (source, analyzer(s), and detector), and data processing methods is presented. Going from the experiment details page to the “Identifications” page on the other hand provides a multi-page list of protein accession numbers identified in the experiment (“Identification List View”). By clicking any of the accession numbers, the user will be taken to the “Identification Detail View.” This page provides several pieces of protein-level information: the originally submitted accession number, along with all the PICR mappings for this protein (inactive accession numbers are shown in a lighter font color). If any mapping was found for the protein, its sequence will be displayed, with the identified peptides highlighted in red. If PTMs are annotated on an identified peptide, they will also be highlighted in yellow there when the corresponding peptide sequence is clicked in the peptide list below.

Next on the page there is a list of all identified peptides for the protein, which will include multiple occurrences of a peptide if it is identified from multiple spectra. If spectra are submitted in the experiment, the spectrum that led to the identification of each peptide can be viewed by following the “View Spectrum Information” link. The PRIDE Spectrum Viewer that displays mass spectra provides the intensity and *m*/*z* for each peak, but also allows the user to perform a manual *de novo* peptide sequencing approach, by using the mass distance between consecutively clicked peaks to assign possible corresponding (modified) amino acids.

## 4 Complex, customized queries on PRIDE: The BioMart interface

BioMart is a query-oriented data management system that does not require any programming knowledge to interrogate, yet allows for very powerful data retrieval 39. The PRIDE BioMart is very popular with users as it gives them complete control about how the data is filtered, and which results are actually retrieved. Additionally, BioMart automatically provides programmatic access through standard web services, which allow developers convenient customized access to the data from software as well. A BioMart is available for many other biological data resources, and an extensive list can be found at http://www.biomart.org. The existence of BioMarts for many different resources, together with the ability to combine two BioMarts in a single query, enables the integration of information across several types of biological data through across-Mart queries.

In the current BioMart interface called BioMart central portal server (http://www.biomart.org/biomart/martview/), it is possible to retrieve data from PRIDE individually, but also to integrate information from PRIDE with other resources. At the time of writing, PRIDE data can be combined with other systems in the following ways:

1. With PRIDE as the main dataset (selected at the top of the BioMart page), integration with Reactome, MSD (protein structures), and the Rat Genome Database is available.
2. PRIDE can also be chosen as the second dataset (at the bottom of the BioMart dataset section) from the resources mentioned in (a), but also from the highly cross-referenced and information-rich UniProt and Ensembl BioMarts.

Through the BioMart, biologists without a bioinformatics background can easily perform complex queries combining PRIDE data with the information present in other important data repositories. The only limitation is that it is not yet possible to combine more than two resources. Additional information on the overall BioMart interface 40 and the specific PRIDE BioMart 36 is also available.

Finally, PRIDE data has also been incorporated into the EBI's new meta search tool (“EB-eye”), accessible at the top from every single EBI page. It allows users to quickly find information based on keywords or protein or gene names or accession numbers across a large variety of EBI resources.

## 5 Submitting data to PRIDE and the PRIDE Converter

The PRIDE repository is entirely dependent on data submissions, as detailed proteomics data cannot be curated from existing literature. Submissions to PRIDE are performed using a publicly available XML data format called PRIDE XML, which is built around the HUPO-PSI mzData standard for MS (http://www.ebi.ac.uk/pride/schemaXmlspyDocumentation.do). And although the PRIDE XML format is well documented, converting proteomics data to this format can be quite challenging. To make submission as easy as possible, different tools for converting existing proteomics data into PRIDE XML have been made available. These include the ProteomeHarvest PRIDE Submission Spreadsheet, which is Microsoft Excel-based (http://www.ebi.ac.uk/pride/proteomeharvest) 36, and the PRIDE Wizard for MASCOT result files (http://www.mcisb.org/resources/PrideWizard) 41. ProteomeHarvest works only on computers running Microsoft Windows, cannot accommodate peak lists, and requires substantial manual effort from the user to populate the Excel sheet. The PRIDE Wizard only accepts MASCOT result files as input. Additionally, both of them were developed for dealing with relatively small volumes of data. Fortunately, the situation has now changed since a new tool called PRIDE Converter has been developed recently 24 (http://code.google.com/p/pride-converter), which is platform independent, and can convert input data from a large variety of popular formats (including MASCOT 42, SEQUEST 43, X!Tandem 44, OMSSA 45, Spectrum Mill, ProteinProphet/PeptideProphet 46, mzXML 47, and mzData), making the submission process much easier and more straightforward, especially for researchers without bioinformatics support (Fig. 1).

**1:**
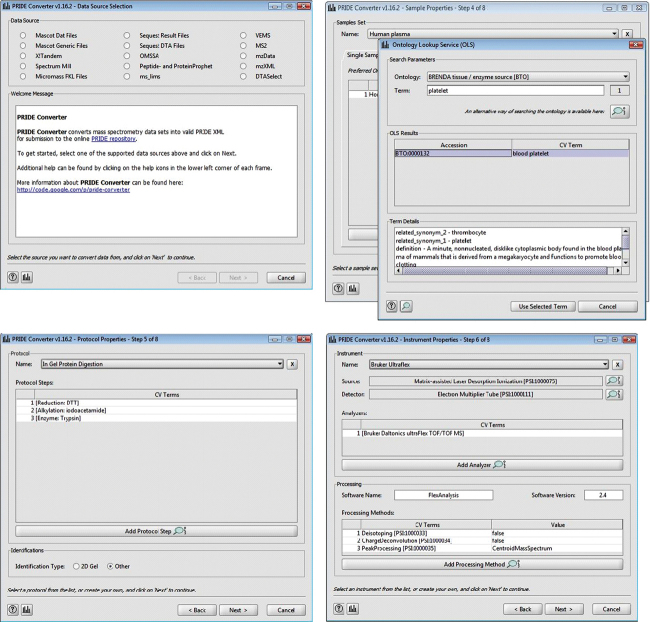
PRIDE Converter application graphical user interface. Screenshots from the PRIDE Converter application. The PRIDE XML file is created by completing eight simple steps. The OLS is used to provide experimental metadata annotation.

It is also possible to submit quantitative data to PRIDE using the PRIDE Converter application for iTRAQ experiments.

Furthermore, certain publicly available proteomics data management tools automatically allow export of data in the PRIDE format 48. Finally, large labs with strong informatics support have also set up a tailored submission pipelines to submit their data to PRIDE. Guidelines have already been published about how to carry out this task 37, but it is always recommended to contact the PRIDE team first if this route is chosen.

Once the PRIDE XML file is created, there are currently two options to submit it to PRIDE:

1. Direct submission *via* the web interface: this submission path is only suitable for small submissions (files up to 15 MB). To make the submission, users will have to be registered in the PRIDE system and log-in, at which point a “Submit Data” option is made available in the left menu on the PRIDE website. It is possible to verify that the files are correctly formatted prior to submission, by validating them against the PRIDE XML 2.1/mzData 1.05 schema *via* the “Validate XML” link in the left menu on the PRIDE site.
2. For larger files, users are advised to first contact the PRIDE team at pride-support@ebi.ac.uk. A curator will then create a private directory for the user in the EBI FTP server and the user will be able to upload their files there confidentially. The restrictions on using the web interface are solely the result of potential problems in the HTTP protocol when uploading large files, as file size limitations are essentially nonexistent for submission purposes. For instance, the largest single PRIDE XML submitted to PRIDE to date was around 86 GB in size.

The overall process of generation, validation, and submission of PRIDE XML files is summarized in Fig. 2. It is important to highlight the PRIDE data policy again here. First of all, data is owned by the original submitter and it will not be changed or reprocessed (note that additional annotations such as the PICR mappings will be added, but these are always clearly flagged as PRIDE additional annotations). Second, data is kept as private by default. It is however easy to get a Reviewer account for privately submitted data. This account can be included in a submitted manuscript and the potential reviewers can thus access the private data in PRIDE anonymously. Data will be made publicly available when the submitter decides to do so (usually after the manuscript has been accepted for publication). Finally, it is also possible to set up “Collaborations” to share private data with selected users. When setting up a collaboration, the submitter remains in control of collaboration membership.

**2:**
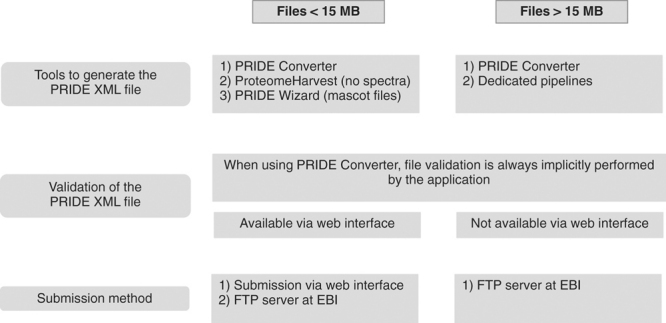
Existing resources for the generation, validation and submission of PRIDE XML files. Schema showing the different tools and resources that can be used to submit data to PRIDE.

## 6 Integration with other repositories

PRIDE is a founding part of the ProteomExchange consortium, together with other important proteomics repositories 49. At this moment, the members of the consortium (PRIDE, NCBI Peptidome, Tranche, PeptideAtlas, and GPMDB) are working on the implementation of a system that will allow the sharing of proteomics data between all the members. Smaller or more specialized repositories will be able to join the consortium as well, and the mechanisms employed to announce new ProteomExchange datasets will be publicly available, allowing any interested party to receive these notifications.

As stated before, each repository has different aims and ways to process or visualize the data. The idea is that submitters to ProteomExchange will be able to get the maximal exposure for their data through a single submission. Within the ProteomExchange consortium, PRIDE and NCBI Peptidome represent the initial submission points since these two repositories do not alter submitted data in any way. As soon as a dataset becomes publicly available, it will be distributed to the other members, and standardized notifications will be made available to the community. Guidelines for ProteomExchange submissions are being finalized, and include three mandatory data types that will have to be included *per* submission: instrument output files (raw data, peak lists), associated metadata, and peptide/protein identifications. Note that this means that not all submissions will qualify to become a ProteomExchange dataset.

However, independent of the ProteomExchange initiative, it has been recently formally agreed that PRIDE and NCBI Peptidome will replicate and share all their data, again making the data even more visible to the scientific community while maximizing the ability of the repositories to capture the data generated in the field.

At present PRIDE users can already benefit from initial data sharing between PRIDE and other repositories. Certain experiments in PRIDE (for instance accession numbers 8172–8544, a ProteomExchange pilot submission) contain links to files stored in the Tranche repository in the “Experiment View” page. For these experiments, it is therefore already possible to get the original raw data or search engine output files, which are not stored as such in the PRIDE system.

Additionally, it is possible to provide links from protein identifications in PRIDE with the corresponding spots in gel images, if these images have been submitted before to World-2D-PAGE 50.

## 7 Future developments in PRIDE

We are currently working on the next iteration of the PRIDE system, which will be version 3. The main changes will include a revised web interface including additional graphical display of information, compliance with the new PSI standards, mzML and mzIdentML (previously known as analysisXML), and an optimized database layout that will ensure fast response times even if the data holdings of the system continue to increase at their current exponential rate. Another innovation will be the ability for submitters, journal editors, and reviewers to request an analysis of a dataset against publication guidelines or minimal reporting requirements such as MIAPE 51. It is important to highlight here that the interaction between PRIDE and the relevant journals will become tighter. As a first step, *Proteomics* is already notifying PRIDE when a manuscript with associated data submitted to PRIDE has been accepted for publication, indicating that the corresponding dataset should be made publicly available.

Another important development, which started in January 2009, is the development of a new database called PRIDE-Q (for Q-rated) that will contain only the highest-quality data from PRIDE. The relationship between the current PRIDE repository and the planned PRIDE-Q resource is very similar to that between UniProtKB/Swiss-Prot and UniProtKB/TrEMBL. The former aims for assured quality and annotation, whereas the latter aims primarily at fully capturing all available data.

The challenging task of quality filtering the data in PRIDE will be carried out in a completely open approach, so feedback from the original submitters and the community at large can be incorporated in iterative refinements of this system.

## 8 Concluding remarks

The PRIDE repository and its satellite tools have been in active use for several years by proteomics researchers. Data submitters have profited from the possibility to keep data access private during peer review of their manuscripts, while allowing journal editors, reviewers, and collaborators to access the data. Data submission to PRIDE has been made much easier, and there is no longer a data size limitation for PRIDE submissions. Researchers can now also retrieve data from PRIDE through various ways, with the BioMart interface as the most versatile of these. Additionally, it is possible to perform across-Mart queries joining PRIDE with other repositories such as Reactome, Ensembl, or UniProt to collect data spanning multiple domains. PRIDE will continue to evolve in lockstep with the field, by implementing emerging standards, setting up data sharing with other repositories, and by creating a new resource called PRIDE-Q, which will contain highly reliable data.
